# Effectiveness of CRSCE-Based De-escalation Training on Reducing Physical Restraint in Psychiatric Hospitals: A Cluster Randomized Controlled Trial

**DOI:** 10.3389/fpsyt.2021.576662

**Published:** 2021-02-16

**Authors:** Junrong Ye, Zhichun Xia, Chen Wang, Yao Liao, Yu Xu, Yunlei Zhang, Lin Yu, Sijue Li, Jiankui Lin, Aixiang Xiao

**Affiliations:** ^1^Department of Nursing Administration, Affiliated Brain Hospital of Guangzhou Medical University (Guangzhou Huiai Hospital), Guangzhou, China; ^2^Department of Traditional Chinese Medicine, Affiliated Brain Hospital of Guangzhou Medical University (Guangzhou Huiai Hospital), Guangzhou, China; ^3^Department of Adult Psychiatry, Affiliated Brain Hospital of Guangzhou Medical University (Guangzhou Huiai Hospital), Guangzhou, China; ^4^Department of Early Intervention, Affiliated Brain Hospital of Guangzhou Medical University (Guangzhou Huiai Hospital), Guangzhou, China; ^5^Department of Cardiothoracic Surgery, Jingzhou Central Hospital, Jingzhou, China; ^6^Department of Intensive Care Unit, West China Hospital of Sichuan University, Chengdu, China

**Keywords:** coercion, de-escalation, physical restraint, psychiatric hospitals, training

## Abstract

**Background:** The use of physical restraint (PR) causes clinical and ethical issues; great efforts are being made to reduce the use of PR in psychiatric hospitals globally.

**Aim:** This study aimed to examine the effectiveness of CRSCE-based de-escalation training on reducing PR in psychiatric hospitals.

**Method:** The proposed study adopted cluster randomized controlled trial design. Twelve wards of a psychiatric hospital were randomly allocated to experimental group (*n* = 6) and control group (*n* = 6). Wards of control group were assigned to routine training regarding PR; wards of experimental group underwent the same routine training while additionally received CRSCE-based de-escalation training. Before and after CRSCE-based de-escalation training, the frequency of and the duration of PR, and the numbers and level of unexpected events caused by PR, were recorded.

**Results:** After CRSCE-based de-escalation training, the frequency (inpatients and patients admitted within 24 h) of and the duration of PR of experimental group, showed a descending trend and were significantly lower than those of control group (*P* < 0.01); compared to control group, the numbers of unexpected events (level II and level III) and injury caused by PR of experimental group had been markedly reduced (*P* < 0.05).

**Conclusions:** CRSCE-based de-escalation training would be useful to reduce the use of PR and the unexpected event caused by PR in psychiatric hospitals. The modules of CRSCE-based de-escalation training can be adopted for future intervention minimizing clinical use of PR.

**Clinical Trial Registration:** This study was registered at Chinese Clinical Trial Registry (Registration Number: ChiCTR1900022211).

## Contribution of the Paper

### What Is Already Known About the Topic?

- Physical restraint is frequently used in psychiatric hospitals, particularly in low and middle income countries (LMICs) and developing countries.
- The use of physical restraint causes severe adverse effects on patients and nurses.
- Some studies reported that, conducting a series of evidence-based approaches helps to minimize the use of physical restraint.

### What This Paper Adds

- This CRSCE-based de-escalation training program is the adaptation of the *REsTRAIN YOURSELF* program in the United Kingdom and the Six Cores Strategies in the United States.
- The frequency of and the duration of physical restraint, and the numbers of injury caused by physical restraint, were remarkably reduced after implementing CRSCE-based de-escalation training program.
- This study demonstrates that, the contents of CRSCE-based de-escalation training program, such as communication skills and humane care service, can be adopted for physical restraint reduction programs.

## Introduction

Physical restraint (PR), the mandatory measure to reduce a patient's physical movement, is applied to stop a patient posing critical risk to others or self (1). In psychiatric hospitals, although PR is regarded as the last resort in emergency, the use of PR is still prevalent with the annual increase. In developed countries, studies exhibited PR frequency in psychiatric hospitals ranged between 3.3 and 34.1% among inpatients, as 2.6% in Norway, 3.1–6.6% in Switzerland, 3.3–8.0% in Germany, 3.8–5.0% in Finland, 5.7% in Wales, 7.3% in England, 11–18% in Italy, and 29.8–34.1% in the United States (2–6). Meanwhile, the clinical use of PR is more common in low and middle income countries (LMICs) and developing countries. Researches showed the PR frequencies were 14.2% in Israel, 23.0% in South Africa, 32.3% in Iran, and 27.2–51.3% in mainland China (4, 7–10). Despite that the reasons accounting for the cross-countries difference remain unclear, the frequently use of PR in psychiatric hospitals has gained public concern worldwide.

The use of PR has caused clinical and ethical dilemma in mental health service, because it results in a wide range of and severe adverse effects on patients and nurses (11). Empirical literatures reported PR caused patients unexpected physical injuries, such as pressure ulcer, increased nosocomial infection, soft tissue injuries, fractures, and even sudden death (12–14). In addition, the clinical use of PR would lead to psychological problems on patients. Investigations found patients undergoing PR experienced the feelings of fear, anger, agitation, depression, anxiety, embarrassment, and PR induced traumatic memories (12, 15–18). PR also causes mental stress among medical personnel, nurses witnessing or conducting PR feel guilty as they believe PR might hurt patients (19–22). In addition, nurses are exposed to critical risk of being assaulted when implementing PR, which might increase their sick-leave from work (23).

Given that the use of PR causes various adverse impacts on patients and nurses, further studies clarify the characteristics of its clinical use. In general, the reasons for using PR are unstoppable violence, suicidal behavior, absconding (discharge without permission), and disturbing behavior (4). Previous studies yielded some demographic factors would be the predictors of using PR, including male, younger age, unemployment or lower income, poor insight, compulsory or voluntary admissions, less outpatient treatment prior to admission, aggressive behavior prior to admission, and being diagnosed with schizophrenia, substance abuse and neurocognitive disorders (2, 4, 5, 7, 9, 24, 25). Besides, the use of PR is also influenced by nurses' attitude, legislative factors, therapeutic environment, and administrative factors (5, 26–30).

To reduce the use of PR and its critical influences on patients and nurses, great efforts has been made to develop PR reduction strategies. The Six *Cores Strategies* are highly acknowledged in restraint reduction program, and have been adapted for different treatment culture in the United States, Spain, Finland, and so on (13, 16, 28, 31–33). In recent years, a newly developed PR reduction program named *REsTRAIN YOURSELF*, is the adaptation of Six Cores Strategies in the North West of England (34). Noticeably, Six *Cores Strategies* and *REsTRAIN YOURSELF* emphasize staff training is one of the most important approaches reducing PR and its adverse effects on patients and nurses. De-escalation techniques, the recommended first-line response to imminent violence, is comprised of self-regulation, communication, risks assessment, actions, and safety maintenance (35, 36). Studies asserted de-escalation techniques training helped to minimized the frequency and duration of PR in psychiatric hospitals (23, 28, 37–39). Being implemented as adjunct intervention, a study by Wale et al. (40) suggested de-escalation training helped to reduce 28.0% of PR episodes, 76.7% of PR duration, and 56.0% of PR caused injuries, respectively. Another research by Duxbury et al. (34), which included de-escalation training that focused on preventing violence in mental health wards, stressed the average reduction of PR by 22% after the intervention period. However, previous studies have some limitations as these studies mainly adopt quasi-experimental design, thus higher level of evidence are needed to confirm the effectiveness of de-escalation training. Besides, the findings of western countries might not be suitable for China due to the difference of medical systems, staffing level, administrative factors, and even social culture. Therefore, by conducting a cluster randomized controlled trial, we examined the CRSCE-based de-escalation training program in reducing PR and its adverse effects on patients and nurses.

## Methods

### Participants and Settings

This study was conducted in a provincial public psychiatric hospital with 24 wards, 1,920 beds. This large-scale psychiatric hospital mainly serves Guangdong, the largest province of southern China with 115 million populations. The ethical approval was obtained from the Institute Review Board (ethical approval number: HAEC-2019-06-K36). This study was registered at Chinese Clinical Trial Registry (registration number: ChiCTR1900022211). The inclusion criteria were: (a) secluded wards for mentally ill patients and; (b) percentage (%) of full-employed nurses of wards > 85. Totally 12 secured wards for mentally ill patients were enrolled. The treatment plan of all patients did not change during study period.

### Trial Design and Randomization

**Figure 1:**
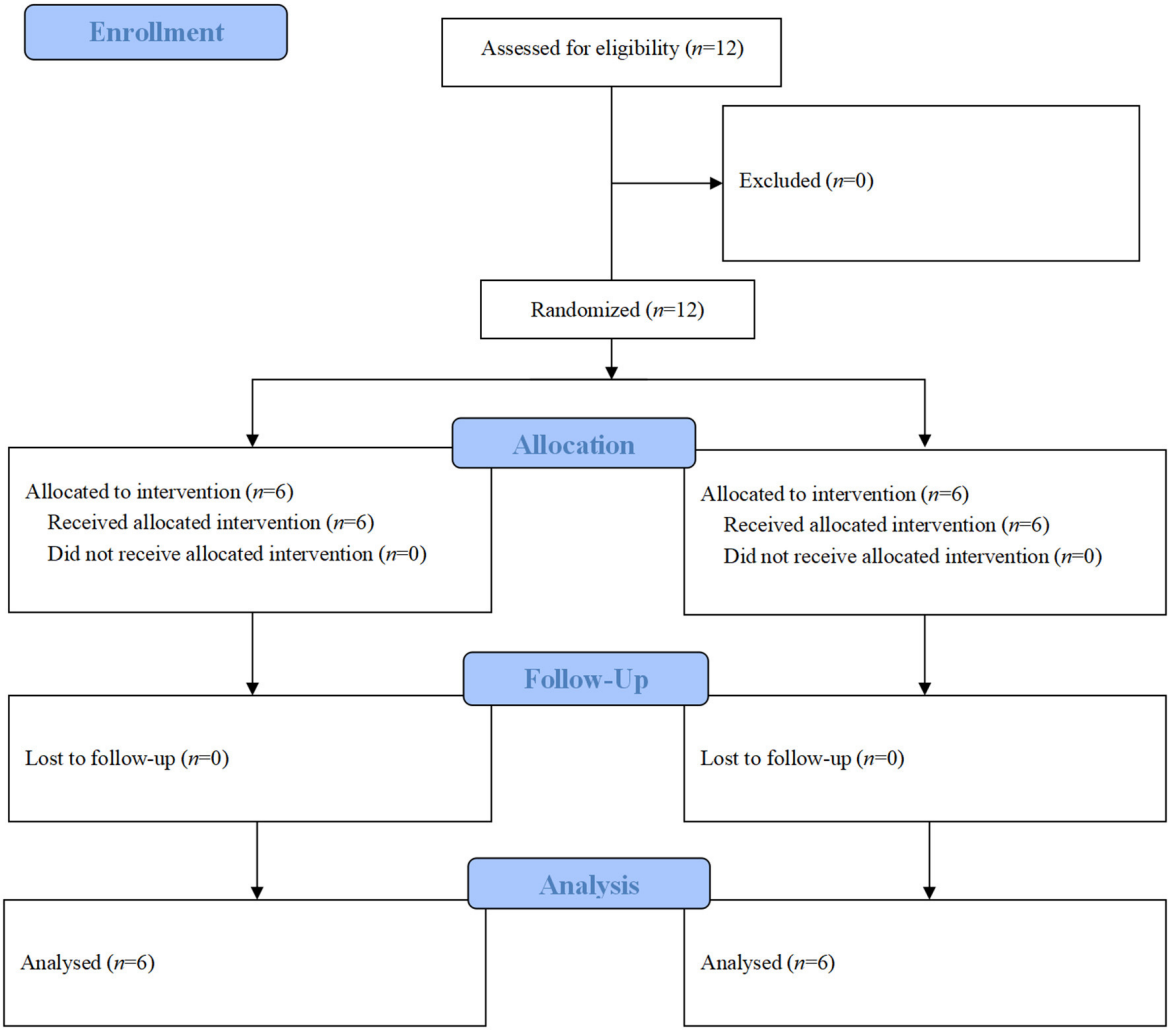
Allocation process of enrolled psychiatric wards.

This study was a two armed, single blinded, cluster randomized, controlled trial. The randomization unit was involved psychiatric wards. By using number generator, recruited wards were consecutively coded and randomly assigned to experimental group and control group according to a 1:1 ratio (Figure 1). The statistician then informed the research coordinator of the group allocations. Afterwards, the training schedule of each ward was designed according to group allocation. To ensure justice, when the experimental group had completed the de-escalation training, and follow-up data had been collected, the control group also received the same de-escalation training.

### Blinding

This was a single blind study. The nursing staff and managers of recruited wards were not aware of group allocation. Data collection was conducted by two research assistants who were not engaged in this study.

### Interventions

Wards of control group were assigned to routine training, which was conducted by training center of sampling hospital; wards of experimental group underwent the same routine training while additionally received CRSCE-based de-escalation training conducted by Huiai Violence Prevention and Management Group (HVPM). CRSCE-based de-escalation training program, aimed to reduce the use of PR, was designed with 5 modules (Communication, Response, Solution-Focused Technique, Care, and Environment). CRSCE training program is consisted of 17 h of lecture learning and 7 h of clinical practice, and would be completed within 1 month. In regard to lecture learning, the lecturers conducted workshop, brainstorm, and demonstrated videos to achieve the learning objects; for clinical practice section, the lecturers organized role play based on real clinical scenarios, and conducted reflective discussion after practicing. The modules, learning objectives, and learning hours of routine training and CRSCE-based training program are presented in Table 1.

**Table 1:** Modules, objectives, and learning hours of routine WPV management training and CRSCE.

**Module**		**Content**	**Objects**	**Hours**		**Routine WPV management training**	**CRSCE training**
				**Lecture**	**Practice**		
Routine WPV management training		Basic Communication Skills of Nursing	To understand the concept of communication skills and its attributes To understand different types of communication skills To learn how to interact with patients in practice	1	–	✓	✓
		Communication Skills in Mental Health Care	To identify the attribute of communication skills in mental health care To distinguish the difference of communication skills between mental health and general nursing To learn how to interact with psychiatric patients	2	1	✓	✓
		Risk Assessment of Violence	To understand the types of WPV To learn how to use different assessment tools To discuss advantage and disadvantage of assessment tools	1	–	✓	✓
		The Ethic and Law in Metal Health Care	To discuss the ethical issues in mental health care To discuss how nurses to balance the ethical issues and law in mental health care	1	–	✓	✓
		De-escalation	To know the concept of De-escalation To identify the attributes of De-escalation To discuss the key components contribute to successful de-escalation	2	–	✓	✓
		Practical WPV coping skills	To learn the breakaway techniques, holding methods To learn the control and restraint methods	2	2	✓	✓
CRSCE	Communication	How to Build the Therapeutic Nurse-Patient Relationship	To identify the factors that influence therapeutic relationship To identify the key components of building the therapeutic relationship with patients To learn how to build therapeutic nurse-patient relationship using communication skills	1	1	**NA**	✓
		The Communication Skills to Aggressive Patients	To learn the communication skills with aggressive patients	1	1	**NA**	✓
	Response	The Early Stage Signal of WPV	To identify the early stage signal of WPV To discuss and learn what a nurse should do when he/she has identified a patient is in WPV early stage	1	1	**NA**	✓
		What Is Your FIRST Reaction When WPV Happens?	To recall nurses' memories of facing violence To share nurses' experience of coping with violence To refresh and discuss the appropriate method to manage WPV	1	–	**NA**	✓
		When WPV Happens, What Should We Do?	To learn how to response WPV To discuss the alternatives of WPV	2	1	**NA**	✓
		What is the Influence of WPV on You?	To discuss and share the influence of WPV on individual	1	–	**NA**	✓
	Solution-Focused Technique	Cognitive Positive Psychology	To learn the concept of cognitive positive psychology and how to use it in clinical work	1	–	**NA**	✓
		The Concept and Principle of Solution-Focused Technique in Nursing	To learn the concept and principle of solution-focused technique	1	–	**NA**	✓
		The Five Stages of Psychological Intervention of Solution-Focused Technique and Its Application	To identify the five stages of psychological intervention of solution-focused technique To learn using the five stages of psychological intervention in practice	2	1	**NA**	✓
	Care	The History and Development of Humane Care Service	To understand the history and development of humane care service To identify the key elements of humane care service	1	–	**NA**	✓
		The Relationship Between Humane Care Service and WPV	To discuss the relationship between humane care service and WPV To learn proving humane care to aggressive patients	1	–	**NA**	✓
		The Humane Care Service in Mental Health Care	To identify the humane care service in mental health care To discuss what nurses can do to provide the humane care service to psychiatric patients	1	1	**NA**	✓
	Environment	The Innovation of Environment in Mental Health Care	To understand the concept of environment in mental health care To understand the change process and development of environment in mental health care	1	–	**NA**	✓
		Evidence Base Practice: The Relationship Between Ward Environment and WPV	To discuss how environment affects the WPV To find out the environmental hazards of WPV through literature review To implement achievable improvement of ward environment	2	1	**NA**	✓
Workplace violence = WPV		CRSCE-based de-escalation training program = CRSCE		Total Learning Hours		**12 h**	**12 + 24 h**
✓: Included modules		**NA**: Not Available					

### Measurements

Initially in trial registration phrase, this study planned to collect the clinical data as primary outcome, and to collect questionnaires reflecting the impacts on nurses as secondary outcome. But the researchers did not have the permission of using questionnaires when this study was started. Finally, this study employed following objective indicators to evaluate the effectiveness of CRSCE-based training program:

a) frequency of physical restraint of inpatients [monthly use of physical restraint of inpatients (%) = monthly number of patient days of physical restraint/total monthly patient days × 100%];
b) frequency of physical restraint in patients admitted within 24 h [monthly use of physical restraint of patients admitted within 24 h (%) = monthly number of patients being physically restrained within 24 h after admission/monthly number of admitted patients × 100%];
c) duration of physical restraint;
  numbers of and severe level (level I: unexpected death; level II: injury need additional medical care; level III: injury did not need additional treatment; level IV: potential injury found by nurses) of accidents caused by physical restraint;
d) numbers of injury of nurses caused by conducting physical restraint.

### Statistical Methods

Data analysis was performed using SPSS 20.0 software. Descriptive data was presented as frequency and percentage if applicable. Quantitative data was reported using means and standard deviations. Student's *t*-test and Chi-square test were adopted to compare group difference. Repeated ANOVA and generalized estimating equation analysis were performed to determine the pre-and-post significant change between groups if applicable. The statistical significance will be set at *P* < 0.05, two tailed, with a 95% confidence interval (CI).

## Results

### Characteristics and Staffing Level of Recruited Department

In total 12 wards were recruited and were randomly allocated to experimental group (*n* = 6) and control group (*n* = 6). There was no group difference in number of bed, turnover rates of bed, number of nurses, and nurse-bed ratio (all *P* > 0.05) (Table 2).

**Table 2:** Characteristics and staffing level of recruited departments (*n*/mean ± sd).

	**Experimental group**	**Control group**	***t/χ*^2^**	***P***
	**(*n* = 6)**	**(*n* = 6)**		
Number of beds	75.00 ± 20.74	86.67 ± 20.90	0.971	0.355
Turnover rate of beds (%)	75.42 ± 17.72	61.74 ± 18.26	−1.317	0.217
Number of patient days				
Before training	69,239	79,069	0.238	0.625
After training	79,663	90,655		
Number of nurses	22.33 ± 2.25	23.50 ± 2.43	0.863	0.408
Nurse-bed ratio (%)	0.31 ± 0.05	0.28 ± 0.04	−1.072	0.309

### Clinical Use of Physical Restraint

**Figure 2:**
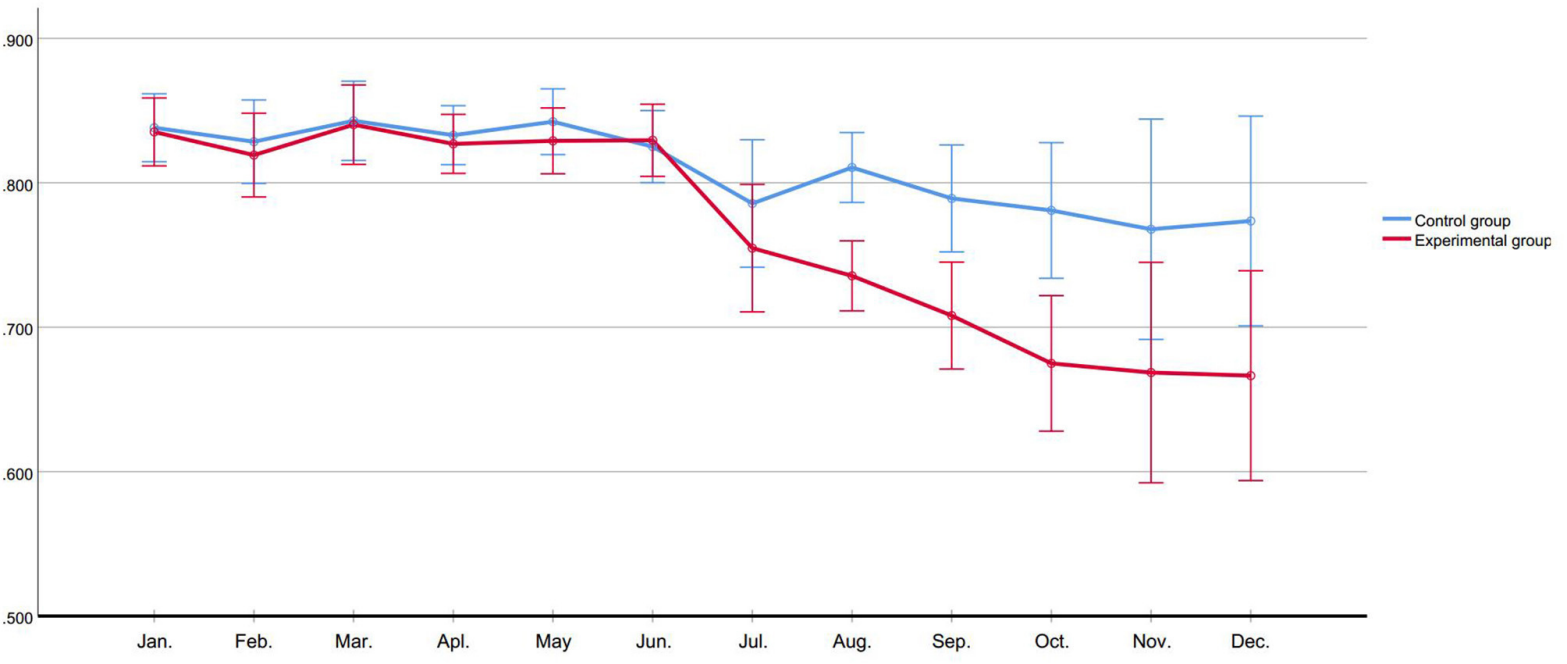
Use of physical restraint of inpatients (%).

**Figure 3:**
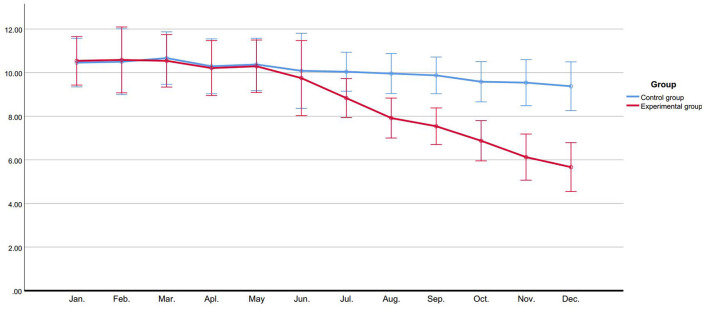
Use of physical restraint in patients admitted within 24 h (%).

We observed the monthly use of physical restraint of inpatients and patients admitted within 24 h, group comparison showed no difference in monthly use of physical restraint in first 6 months. After de-escalation training, in experimental group, the monthly use of physical restraint of inpatients (*F*_time_ = 3.651, *P* < 0.001) and patients admitted within 24 h (*F*_time_ = 3.500, *P* < 0.001) showed a descending trend; and compared to control group, the monthly use of experimental group of physical restraint of inpatients (*F*_group_ = 5.374, *P* = 0.043) and patients admitted within 24 h (*F*_group_ = 12.065, *P* = 0.006) were significantly lower (Figures 2, 3).

### Duration of Physical Restraint

**Figure 4:**
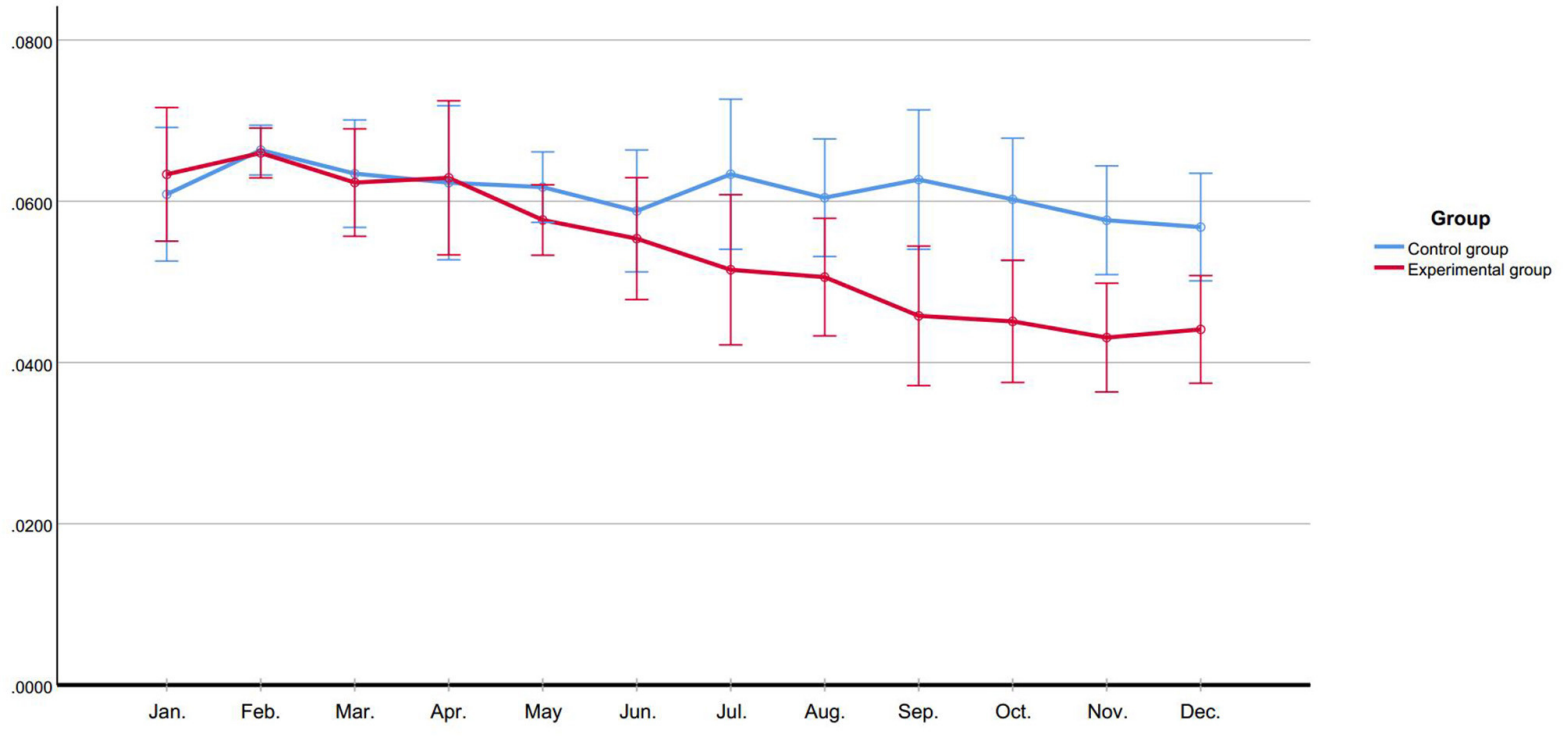
Duration of physical restraint (hour).

In first 6 months, the average duration of physical restraint did not show a significant group difference. After de-escalation training, the average duration of physical restraint of experimental group presented a decreasing trend (*F*_time_ = 9.174, *P* < 0.001) and was remarkably lower than control group (*F*_group_ = 5.054, *P* = 0.048) (Figure 4).

### Number of Different Level of Accidents Caused by Physical Restraint

The total numbers of accidents caused by physical restraint before and after de-escalation training did not exhibit significant group difference (χ^2^ = 1.067, *P* = 0.301) (Table 2). Further generalized estimating equation analysis showed, after de-escalation training, the numbers of level II and level III accidents of experimental group had reduced, the constituent ratio of different levels of accidents showed significant changes before and after de-escalation training (β = 0.503, 95% *CI* = 0.027–0.980, *P* = 0.038) (Table 3).

**Table 3:** Numbers of accidents caused by physical restraint and numbers of injury of nurses caused by conducting physical restraint.

	**Numbers of accidents caused by physical restraint**										**Numbers of injury of nurses**			
	**Before training**				**After training**				***χ^2^***	***P***	**Before training**	**After training**	***χ*^2^**	***P***
	**II**	**III**	**IV**	**Total**	**II**	**III**	**IV**	**Total**						
Experimental group (*n* = 6)	5	12	7	24	0	4	9	13	1.067	0.301	15	4	4.184	0.041
Control group (*n* = 6)	6	13	10	29	4	12	11	27			13	16		

### Numbers of Injury of Nurses Caused by Conducting Physical Restraint

After de-escalation training, the number injury of experimental group was apparently lower than control group (χ^2^ = 4.184, *P* = 0.041) (Table 3).

## Discussion

This study evaluated the effectiveness of CRSCE-based de-escalation training program on reducing PR in a public psychiatric hospital. The findings of this study asserted enhancing psychiatric nurses' competence of de-escalating conflict helped to decrease the clinical use of PR. This study adopted the frequency and duration to evaluate the clinical use of PR. After the CRSCE-based de-escalation training, remarkable declines were witnessed in the frequency and duration of PR in experimental group (all *P* < 0.05). This implied the context of CRSCE-based de-escalation training program would be effective in reducing PR. Overall, empirical findings support that de-escalation training is one of the vital adjunct measures of reducing the use of coercion in psychiatric hospitals. Duxbury et al. (34) reported *REsTRAIN YOURSELF* program lead to the significant reductions (range between 18.8 and 64.7%) in the restraint rates among five psychiatric hospitals. A randomized control trial by Putkonen et al. (31) reported, by implementing Six *Cores Strategies* of reducing PR, the proportion of patient-days with coercion declined from 30 to 15%. In addition, Wale et al. (40) and Putkonen et al. (31) highlighted that evident reduction of seclusion-restraint time was seen after conducting Six *Cores Strategies*. It is noticeable that *REsTRAIN YOURSELF* program is developed from Six *Cores Strategies*, thus the intervention of both program is similar. However, the findings above should be interpreted with cautions because this study only explored the effectiveness of de-escalation training.

Literature reviews by Price et al. (23) and Gaynes et al. (38) proposed, more evidence was needed to decide the effectiveness of de-escalation training in decreasing the use of physical restraint. A recent study by Haefner et al. (41) yielded, a quality improvement project adopting de-escalation had reduced the seclusion rate from 5.9 to 4.4% (*P* = 0.349). The outcome of our study showed, after CRSCE-based de-escalation training program, both the frequency of PR and the duration of PR were significantly cut down due to context of proposed program. On one hand, enhancing the competence of de-escalating might help nurses to build the appropriate nurse-patient relationship and to deal with the conflict by using solution-focused technique, thus conflicts might be solved in the early stage. On the other hand, the contents of CRSCE-based de-escalation training program were expected to guide nurses to rationally conduct PR and to reduce unnecessary PR, by which the duration of PR would be shortened.

In addition, the finding of this study is worth to be compared with the outcome of national 686 project. Overall, both proposed study and the 686 project aim to eliminate coercion toward patients. The 686 project, first national commitment focusing on rebuilding public mental health service, was launched in December 2004 (42). Before the 686 project, a great number of severe psychiatric patients in rural area were unable to obtain mental health service and were long-term locked at home. The 686 project established multifunctional treatment teams (MTT) of well-trained mental health specialists, and assigned MTT to community to directly provided routine follow-up, community-based care, and necessary treatment at no cost. In consequence, the number of patients who were long-term restrained at home had been crucially reduced (42). The 686 project has not covered the institutional use of physical restraint, but it is equally important to discuss the frequently use of physical restraint in psychiatric hospitals. Previous studies of Chinese psychiatric hospitals reported approximately 27.2–51.3% of psychiatric inpatients had ever been physically restrained (7, 9), which was much more frequently than the results of western countries. However, this study focuses on reducing the use of PR in psychiatric hospitals. The results of this study propose, conducting de-escalation training program is a possible approach of minimizing the frequency of and the duration of PR, as well as the PR induced unexpected events.

The use of PR leads to unexpected events to patients and nurses (1), but very few studies of PR reduction program simultaneously observed the accident caused by PR. This trial included the numbers and levels of accident caused by PR as secondary outcome assessing CRSCE-based de-escalation training program. After the intervention, despite no significant group difference in total numbers of accident was found (*P* > 0.05), the proportion of level II and level III accident of experimental group were statistically lower than those of control group (*P* < 0.05), proposing de-escalation training should be a useful approach of reducing injury caused by PR. Besides, the numbers of injury of nurses during implementing PR was significantly reduced after de-escalation training, this might be associated with the decrease of PR use.

In general, studies evaluating PR reduction programs have some limitations. The variation between wards is a major concern when examining the effectiveness of PR reduction program, because the variables regarding the characteristics of recruited wards might potentially influence the PR use (34). This cluster randomized controlled trial took account of variables measuring the ward scale (number of bed), bed turnover rate, and staffing level (number of nurses, and nurse-bed ratio). No significant difference of characteristics of recruited wards were found at baseline, such acceptable homogeneity might due to the fact that all recruited wards were from one psychiatric hospital. Despite the success in reducing the use of PR, it should be noted that the recruited wards were in large scale, high bed turnover rates, and low staffing level. Thus, our findings are expected to be meaningful in LMICs and other developing countries where psychiatric hospitals have similar characteristics.

To concluded, implementing CRSCE-based de-escalation training program was helpful to reduce the clinical use of PR, as well as to reduce its adverse impacts on patients and nurses. CRSCE-based de-escalation training program included comprehensive contents of coping with violence, which was expected to be useful and feasible to psychiatric wards.

## Strengths and Limitations

The major strength was, the cluster randomized, controlled trial design was used to evaluated in this study. In addition, this study observed the indicators that reflecting the results of conducting PR (the number of accidents caused by PR and the numbers of injured nurses because of conducting PR), which were rarely reported in previous studies. This study has following limitations: (a) Despite this study included objective clinical indicators as outcome measurement, the before-and-after evaluation on nurses was not observed; (b) the effectiveness of CRSCE-based de-escalation training program was examined in one psychiatric hospital, which might influence the generalization of conclusion; (c) a self-developed, Chinese version handbook was used for CRSCE-based de-escalation training program, but these materials are temporarily for internal use. However, the key contents of CRSCE-based de-escalation training program has been presented which would help develop the de-escalation training program in other cultural contexts.

## Data Availability Statement

The datasets presented in this article are not readily available because we will share data for co-operation only. Requests to access the datasets should be directed to 543061910@qq.com.

## Ethics Statement

The studies involving human participants were reviewed and approved by Institute Review Board of Guangzhou Medical University (ethical approval number: HAEC-2019-06-K36). Written informed consent for participation was not required for this study in accordance with the national legislation and the institutional requirements.

## Author Contributions

JY, ZX, and AX conceived this study. JY, ZX, and YL participated in sampling methods design. YX and YZ participated in data statistical analysis. ZX, LY, SL, and JL engaged in data collection. JY, CW, and ZX drafted the manuscript. All authors contributed to the article and approved the submitted version.

## Conflict of Interest

The authors declare that the research was conducted in the absence of any commercial or financial relationships that could be construed as a potential conflict of interest.
